# MultiMetEval: Comparative and Multi-Objective Analysis of Genome-Scale Metabolic Models

**DOI:** 10.1371/journal.pone.0051511

**Published:** 2012-12-14

**Authors:** Piotr Zakrzewski, Marnix H. Medema, Albert Gevorgyan, Andrzej M. Kierzek, Rainer Breitling, Eriko Takano

**Affiliations:** 1 Department of Microbial Physiology, University of Groningen, Groningen, The Netherlands; 2 Groningen Bioinformatics Centre, University of Groningen, Groningen, The Netherlands; 3 Faculty of Health and Medical Sciences, University of Surrey, Guildford, Surrey, United Kingdom; 4 Institute of Molecular, Cell and Systems Biology, College of Medical, Veterinary and Life Sciences, University of Glasgow, Glasgow, United Kingdom; Virginia Commonwealth University, United States of America

## Abstract

Comparative metabolic modelling is emerging as a novel field, supported by the development of reliable and standardized approaches for constructing genome-scale metabolic models in high throughput. New software solutions are needed to allow efficient comparative analysis of multiple models in the context of multiple cellular objectives. Here, we present the user-friendly software framework Multi-Metabolic Evaluator (MultiMetEval), built upon SurreyFBA, which allows the user to compose collections of metabolic models that together can be subjected to flux balance analysis. Additionally, MultiMetEval implements functionalities for multi-objective analysis by calculating the Pareto front between two cellular objectives. Using a previously generated dataset of 38 actinobacterial genome-scale metabolic models, we show how these approaches can lead to exciting novel insights. Firstly, after incorporating several pathways for the biosynthesis of natural products into each of these models, comparative flux balance analysis predicted that species like *Streptomyces* that harbour the highest diversity of secondary metabolite biosynthetic gene clusters in their genomes do not necessarily have the metabolic network topology most suitable for compound overproduction. Secondly, multi-objective analysis of biomass production and natural product biosynthesis in these actinobacteria shows that the well-studied occurrence of discrete metabolic switches during the change of cellular objectives is inherent to their metabolic network architecture. Comparative and multi-objective modelling can lead to insights that could not be obtained by normal flux balance analyses. MultiMetEval provides a powerful platform that makes these analyses straightforward for biologists. Sources and binaries of MultiMetEval are freely available from https://github.com/PiotrZakrzewski/MetEval/downloads.

## Introduction

Living cells owe their existence to complex metabolic networks, in which large numbers of chemical conversions occur to allow the cells to harvest energy, sustain themselves and reproduce. In the past decades, methodologies have been developed to systematically describe and quantitatively analyse (parts of) the metabolic network of a cell in computational models [1], [2]. Such reconstructions have already been of great use to develop a better understanding of the metabolic architecture and dynamics of various organisms [3], [4].

Genome-scale constraint-based metabolic models are reconstructions of metabolism that comprise the stoichiometries of all reactions predicted from whole genome sequences based on the presence of enzyme-coding genes. Accordingly, they can be used to model the steady-state behaviour of the metabolism of a whole organism [5], [6]. Well-accepted procedures on how to generate genome-scale constraint-based models are available, based on Enzyme Classification annotations and generic gap-filling procedures [7].

The resulting metabolic models can be used to perform several kinds of analyses [8], [9], the most popular one being flux balance analysis (FBA) [10]. In this method, the fluxes of metabolites through the network are calculated based on the stoichiometry of each reaction and an objective function that specifies for which goal (e.g. maximization of biomass production from a given input or minimization of nutrient uptake) the fluxes are optimized.

Recently, high-throughput methods have been developed to generate and gap-fill metabolic models for multiple species in a rapid and standardized way [11], based on genome annotations obtained with a uniform method. Even though the resulting models still need to be compared with experimental data to achieve optimal quality [12] and the gap-filling implemented by SEED is not always optimal [13], [14], automatically generated models that have undergone a limited amount of manual curation are already useful for obtaining a rough assessment of the metabolic capabilities of cellular systems.

The standardization offered by automation opens up the road for comparative modelling, as little model reconciliation is needed, in contrast to what is usually the case for manually reconstructed models [15]. Comparative analysis of genome-scale metabolic models is an intriguing new field with diverse potential applications [16], [17]. For example, it can be used to detect evolutionary differences between metabolic networks of related species and predict their relative adaptive ecological value [18]. It can also be used to assess the suitability of a range of species for a particular biotechnological application (e.g., biofuel or drug production) based on the topologies of their metabolic networks, which could then inform the choice of industrial production hosts [19].

As well as studying multiple models at the same time, it can also be very revealing to optimize models for multiple objectives simultaneously [20], [21]. Many different ‘natural’ objective functions have been proposed, such as maximization of biomass, secondary metabolite production or ATP production, minimization of total flux, minimization of redox potential, and minimization of nutrient uptake [22]. For most of these, there are reasons to believe that the cellular flux distribution can be expected to have evolved in a way that optimizes the objective, at least under specific conditions. It can even be argued that evolution has driven biological systems toward an optimal compromise between all of these, sometimes conflicting, objectives. Other relevant objective functions that one would like to consider are those that correspond to the aims of bioengineering instead of evolution, such as the maximization of the production of a specific metabolite. Unfortunately, as implementing different objective functions is relatively difficult in most existing analysis platforms, many published studies have been restricted to exploring a single objective function, usually maximization of biomass production (although interesting studies have been performed that explore different objective functions, e.g. [23], some of which have been made available through the COBRA toolbox [24]).

A pair of objective functions (such as a biomass objective function and the objective function of production of a specific compound) can be balanced to find the so-called Pareto front [25] between the two objectives. The Pareto front comprises the set of “Pareto-optimal” solutions, for which one objective can only be improved at the expense of the other objective. Bacterial metabolism has recently been shown to operate close to such Pareto fronts [26]. An analysis of such a front enables one to predict the interactions between different metabolic processes and priorities within the cell. For example, one can identify the extent to which two objectives compete for the use of the same enzymatic pathways. Moreover, one can use the results to predict the balance between the objectives that is optimal for sustaining biomass levels while producing as much of a certain valuable metabolite as possible.

Here, we describe a new software package, Multi-Metabolic Evaluator (MultiMetEval), a simple framework that provides an efficient and user-friendly interface for the comparative study of multiple models and the use of multiple objective functions. The software has been conveniently linked up to the SurreyFBA package for metabolic modelling [27], allowing for easy interaction with general modelling algorithms. In order to make the tool widely useful, it includes a new global SBML Level 2 parser that enables input of models from popular modelling platforms, including SEED [11], [28], KGML [29] and COBRA [30], overcoming previous compatibility issues between different SBML flavours that severely impaired comparative analyses. Moreover, all functionalities are organized in a graphical user-interface that allows the user to quickly generate publication-quality plots from the results and export the results for downstream analyses in other software packages.

In a case study, we show how the principles of comparative modelling can be applied to a concrete biological problem with our software, in a comparative study of the metabolic networks of 38 actinobacteria. Based on the 38 genome-scale models, we predict the suitability of different bacterial strains for the heterologous production of a range of different secondary metabolites and use multi-objective analysis to study the dynamic balance between the biomass objective and the compound production objective. We find that the maximally attainable fluxes to a natural product vary greatly between species as well as between the chemical classes of compounds. Moreover, we observe discrete switch-like behaviour in the models when the priority of the compound production objective function is gradually increased compared to the biomass objective function; this provides a possible systems-level explanation for the metabolic switches observed in the onset of secondary metabolism in such organisms [31].

### Design and Implementation

The MultiMetEval comparative analysis framework was written in Java 6 Standard Edition with an interface handled by the Swing framework and integrated plot generation handled by the JFreeChart library. It is functional in both Windows and Linux operating systems. The program was built upon the SurreyFBA framework [27], which is used as an engine for the basic FBA calculations. Additionally, in order to read input models from a large range of sources (e.g. SEED [11], [28] and COBRA [30]), a Python-based universal SBML parser was generated to convert input SBML files into a valid SurreyFBA input format. Combined with the parser and the SurreyFBA engine, MultiMetEval allows for high-throughput comparative and multi-objective analysis of metabolic models that share the same syntax.

### Parsing of Input SBML Files

Incompatibility of SBML models coming from different frameworks has been a major drawback for comparative studies [15]. SBML Level 2 itself is a general-purpose language for systems biology, and can be used for storing a great number of data types. There is, however, still no universally adopted definition of FBA-specific parameters within the SBML namespace. Therefore, gene-protein-reaction association rules and reaction capacity bounds have to be defined using annotations and general parameters. This leads to many different format varieties of SBML, in which the data relevant for FBA are stored in different ways.

Existing FBA frameworks make use of their own parsers enforcing usage of their own SBML format variety. In order to make SBML files from different frameworks cross-compatible in our tool, we generated a parser that can convert any major SBML format variety into the SurreyFBA format. As we show in **Table 1**, our parser adds a flexibility that has not been possible in the other major FBA tools. In principle, our parser could easily be implemented in other contexts as well.

**Table 1:** Comparison of parsing capabilities of MultiMetEval with other FBA frameworks.

Framework	SEED-generated SBML	KGML-derived SBML	COBRA-generated SBML
MultiMetEval	+	+	+
COBRA	–	+/−	+
VANTED	–	+	–
SurreyFBA 1.0	–	+/−	+

Table showing SBML parsing abilities of the most popular FBA tools. Only the MultiMetEval parser is able to successfully process SBML models from SEED [11], [28], KGML [29] and COBRA [30].

### Comparative Analysis

MultiMetEval provides a user-friendly facility to perform comparative analysis of multiple metabolic models, by combining batch runs of the single model analysis functionalities provided by SurreyFBA with new features that allow for convenient multi-model input and output.

The basic units analysed by the comparative analysis module are “model collections”, which are sets of models selected by the user for analysis and parsed into the same format by the universal SBML parser.

A specific menu allowing user-friendly construction of such collections is available via the File menu. To allow reuse of models in different collections, the collections can be created as subsets of a main model repository that holds all models that were imported to the program. Models can easily be added to the main repository and then moved to any collection in the same window.

**Figure 1:**
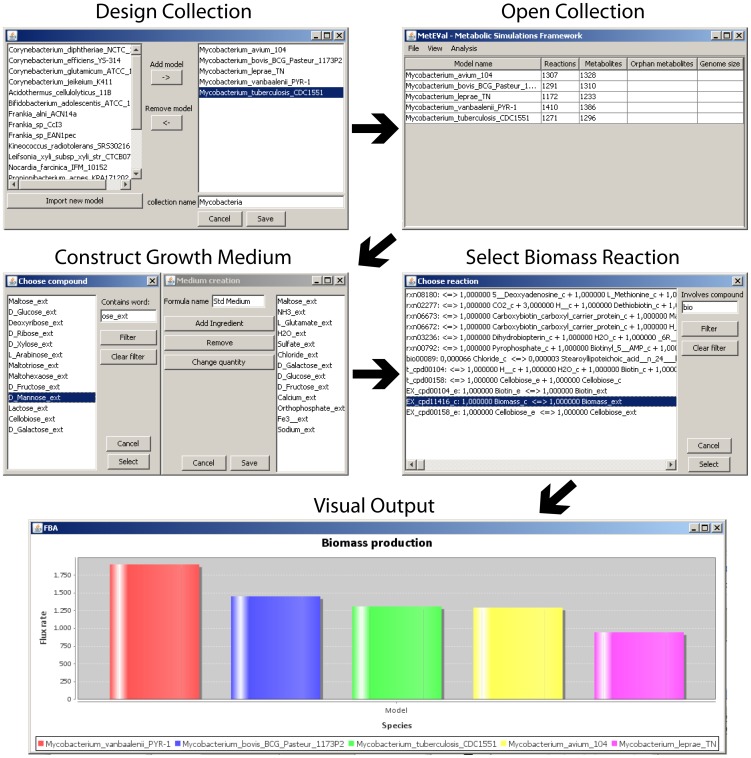
Workflow of comparative metabolic analysis in MultiMetEval.

For every model, the number of reactions, metabolites, orphan reactions and orphan metabolites are detected and displayed in MultiMetEval’s main overview table when a collection is opened. FBA can be performed on the entire collection at once by clicking a simple menu button, and results are output in a single spreadsheet table (**Figure 1**).

Our framework also offers a growth medium editor, which allows comparative analysis not only of different models, but also in different growth conditions. In order to make sure that the medium is compatible with the models, the medium description format used in our framework operates only on the metabolites present in a given collection and restricts the choice of medium ingredients to those which were defined in any of its models as external. The motivation for this is, of course, that these are the only metabolites that can be consumed by at least one model.

### Multi-objective Analysis by Pareto Front Calculation

MultiMetEval allows performing multi-objective analysis by calculating the Pareto front [20], [32] for maximization of two given reactions. Compared to the weighted sum approach (which was already implemented in SurreyFBA), Pareto front calculation is more informative, as it avoids the arbitrary nature of weight assignment.

In this analysis, MultiMetEval calculates the tradeoff between two objectives. Often, the first objective will be the biomass production reaction, but, in principle, MultiMetEval can calculate a Pareto front for the optimization of any combination of two fluxes of reactions that co-occur in the same model.

In the Pareto front calculation, first the maximal possible flux of the first objective is calculated. This value will be used in the following steps as a constraint that is iteratively decreased at each step. So after calculating the maximal flux of objective one, the program will carry out optimizations for the second objective *n* times (were *n* represents the resolution), and with each simulation step the constraint put on the reaction by the first objective will decrease unless its value reaches zero.

**Figure 2:**
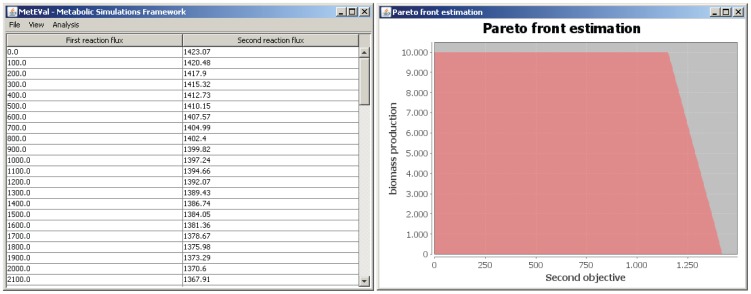
Table and plot output from the Pareto front calculation routine. The first steps are identical to those in Figure 1, except that only one organism is selected and two reactions are selected to calculate their trade-off.

The results of the multi-objective analysis are output to a results table as well as in a visual plot (**Figure 2**).

In addition to the implementation of the Pareto trade-off routine in the MultiMetEval framework described here, we also implemented it in the SurreyFBA command-line interface as well as in JyMet, the single-model analysis framework from SurreyFBA [27], for additional flexibility.

## Results and Discussion

Comparative and multi-objective metabolic modelling has many exciting applications in systems and synthetic biology [4], [33], [34]. To illustrate the power of these approaches, we applied the MultiMetEval tools in an exemplary case study on the production of secondary metabolites in actinobacteria. We show how comparative FBA can be used to identify differences between organisms in their theoretical production capacities for such metabolites, as well as differences in the extent to which biomass production competes with secondary metabolite biosynthesis.

### Comparative FBA of Secondary Metabolite Biosynthesis by 38 Actinobacteria

In our comparative FBA analysis, we constructed a model collection in MultiMetEval from the 38 genome-scale metabolic models of actinobacteria that were recently constructed and curated by Alam et al. [17] (excluding the two *Tropheryma* models, but including models for *Bifidobacterium adolescentis* ATCC 15703, *Bifidobacterium longum* NCC2705 and *Kineococcus radiotolerans* SRS30216). We then reconstructed biosynthetic pathways for 15 secondary metabolites of different classes that were present as annotated pathways in the KEGG database. These included polyketides (erythromycin, tylosin, aureomycin, tetracycline), aminoglycosides (butirosin, neomycin, streptomycin), aminocoumarins (clorobiocin, coumermycin, novobiocin), nonribosomal peptides (enterobactin, pyochelin, cephalosporin, penicillin) and a beta-lactam (clavulanic acid). Such types of compounds are highly relevant biotechnologically, because they often have antimicrobial or anti-cancer activities [35]. Their biosynthetic pathways can be (re-)engineered with synthetic biology approaches and expressed for purposes of drug discovery and industrial production [36]. For each of the 15 metabolites, derivative models were then made for all 38 actinobacteria, in which the biosynthetic pathway for the metabolite was added to the genome-scale model. For all 38 • 15 = 570 models, FBA was then performed using MultiMetEval on a minimal medium with equal amounts of glucose as the sole carbon source, ammonium as the sole nitrogen source, and orthophosphate as the sole phosphorus source. The cellular objective was maximization of the production of the secondary metabolite. A limited number of (maximally seven) reactions for glucose uptake and methionine biosynthesis, as well as compound-specific reactions for precursor biosynthesis were added to each model to enable it to produce the compound on the minimal medium (see Table S1).

**Figure 3:**
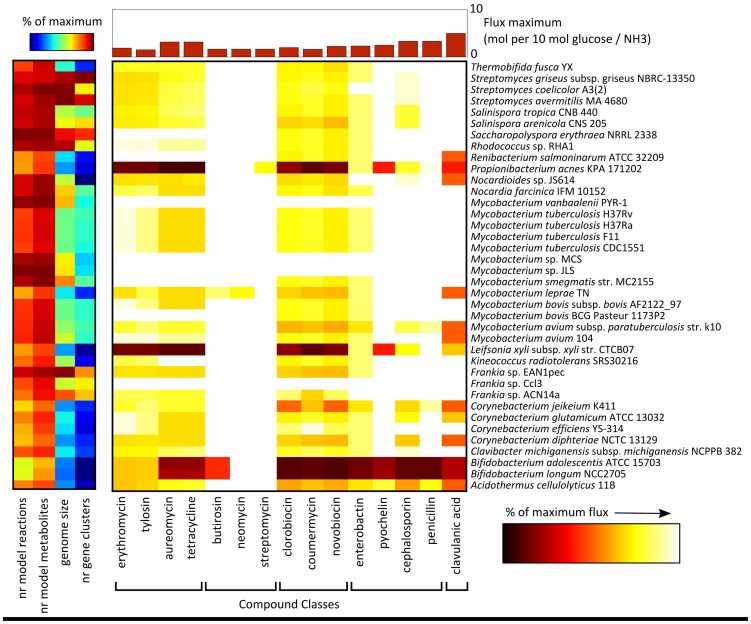
Theoretical maximum fluxes of secondary metabolite production. The heat map shows relative maximal fluxes of the final biosynthetic step in the metabolic pathways leading 15 different secondary metabolites, which were incorporated into the genome-scale metabolic models of 41 actinobacteria. Flux balance analysis was performed on the minimal medium described by Alam et al. [17]. White indicates a high relative flux level, red indicates a low relative flux level (as % of the maximally obtained value across all species, displayed at the top of the figure). In the heatmap on the left, the number of model reactions and metabolites, the genome sizes and the number of secondary metabolite biosynthesis gene clusters (predicted using antiSMASH [54]) are plotted.

**Figure 3** shows the resulting heat map representing the theoretical maximal production rates of the 15 secondary metabolite classes in all 38 actinobacteria. The intensity of a colour depicts the relative flux rate – the lighter the colour (closer to white), the higher a given flux value is in comparison to others from the same column.

As no regulatory and kinetic information is used in the constraint-based models, one should note that the variation observed between the species only represents the difference due to differences in network topology given the medium composition used. Still, it is intriguing that substantial differences in theoretical production capacities are observed between the actinobacterial species. As expected, we observe some correlation with general topological properties of the metabolic networks such as the numbers of reactions and metabolites: minimalistic genomes generally tend to be less efficient predicted production hosts (e.g., *Bifidobacterium* and *Propionibacterium*). However, these differences clearly do not account for all the variation observed. Among the most interesting exceptions is the severely genome-minimized *Mycobacterium leprae*, which still reaches surprisingly high predicted fluxes. Members of the same class of secondary metabolite (which also have similar precursors) are usually predicted to be most efficiently produced in the same hosts. An exception is formed by two nonribosomal peptides, cephalosporin and enterobactin, for which fewer species are able to obtain the maximum observed flux towards compound production than for two other nonribosomal peptides, pyochelin and penicillin. This is probably due to the requirement of additional precursors, 2-oxoglutarate and 2,3-dihydroxybenzoic acid, respectively, for these two molecules, which are not required for penicillin and pyochelin.

When we investigated the presence of which reactions influence fluxes most, by calculating the correlation between reaction presence/absence and maximum fluxes for each compound (Table S2), we could observe that in at least a number of cases this corroborated current biochemical knowledge. For example, clavulanic acid fluxes most strongly correlate with the presence of a reaction (rxn00101) to convert urea into CO_2_ and NH_3_, which corroborates the unusual presence of a microbial urea cycle in its native host organism *Streptomyces clavuligerus* [37], [38]. Also, the fluxes towards several compounds (the macrolides, aminocoumarins and pyochelin) correlated with the presence of a reaction (rxn00141) converting S-adenosylhomocysteine to adenosine and homocysteine, which corroborates evidence for a positive effect of S-adenosylmethionine regeneration on antibiotic biosynthesis [39].

Interestingly, the fact that the genome of a species has a lot of secondary metabolite biosynthetic gene clusters does not necessarily mean that its metabolic network is optimized for a higher production of such metabolites compared to other species: high metabolite diversity does not imply high metabolite production titers. The fact that streptomycetes, such as *Streptomyces coelicolor* and *Streptomyces avermitilis*, famous for their production of a wide variety of clinically and biotechnologically important secondary metabolites [40], [41] and endowed with about 25–30 gene clusters per genome, do not score particularly highly suggests that their metabolic networks may not have been optimized for achieving high production titers of such metabolites during their evolution. This may partly explain why extensive metabolic engineering and classical strain optimization have usually been essential to optimize production strains for economically viable metabolite production, often with tremendous improvements in titres [42]–[44].

On the other hand, models representing species from the taxonomic branch of free-living mycobacteria (*Mycobacterium vanbaalenii*, *Mycobacterium* sp. MCS, and *Mycobacterium* sp. JLS) achieve the highest predicted production rates for secondary metabolites in the simulations, although they have only about 15 secondary metabolite gene clusters per genome. The difference with the pathogenic *Mycobacterium* species, such as *M. tuberculosis*, *M. bovis* and *M. leprae*, may be explained by the further genome minimization of the pathogenic species, which may have led to a loss of flexibility in the metabolic networks and consequently an increase in pathway competition.

Generally, comparative modelling as described here could lead to a more systematic approach towards the identification of suitable “universal hosts” for heterologous expression of gene clusters [47]–[49]. Specifically, this preliminary analysis already suggests that free-living mycobacteria might be an attractive starting point for the generation of a minimal actinobacterial genome for use in synthetic biology approaches [45], [46], especially as all three of them belong to the fast-growing mycobacteria.

As expected, similar patterns of theoretical maximal production rates across organisms were observed for compounds with similar chemical structures, such as the aminocoumarins novobiocin, coumermycin and clorobiocin. Also notable is that the metabolic networks of some organisms appear more fit for the production of certain compounds than others. For example, *Renibacterium salmoninarum* ATCC 32209 is predicted to be one of the best producers of polyketides and one of the worse producers of clavulanic acid. This suggests that the species differences observed are not caused by the presence or absence of single enzymes, but that different factors play a role for different compound types. Some aspects that could play a role are 1) the presence or absence of pathways directed towards the necessary precursors (metabolic detours are probably energetically costly), 2) efficiency of ATP generation from the used carbon source glucose, and 3) the ability of models to re-utilize the (sometimes quite exotic) side products of biosynthetic pathways to generate more precursors.

Of course, it should be kept in mind that this study used only mildly curated automatically generated metabolic network models to illustrate the main concepts of comparative flux balance analysis, and a more careful manual curation will be needed before committing substantial experimental resources to testing the hypotheses suggested here. Additionally, more systematic analysis of the specific differences between topologies associated with high and low production capacities of the different compound types may offer specific leads for metabolic engineering, by revealing topological bottlenecks. Another interesting follow-up study would consist of designing several additional media to study the dynamic interactions between network topology and medium composition or environmental niche.

### Analysis of the Trade-off between Secondary Metabolite Biosynthesis and Biomass Production

Biotechnological optimization of natural product biosynthesis often suffers from pathway competition with fluxes leading to the synthesis of biomass components [50], [51]. In order to assess competition between secondary metabolite biosynthesis and biomass production for selected key species and metabolites, we used multi-objective analysis to calculate Pareto fronts between the biomass objective and the compound production objective.

**Figure 4:**
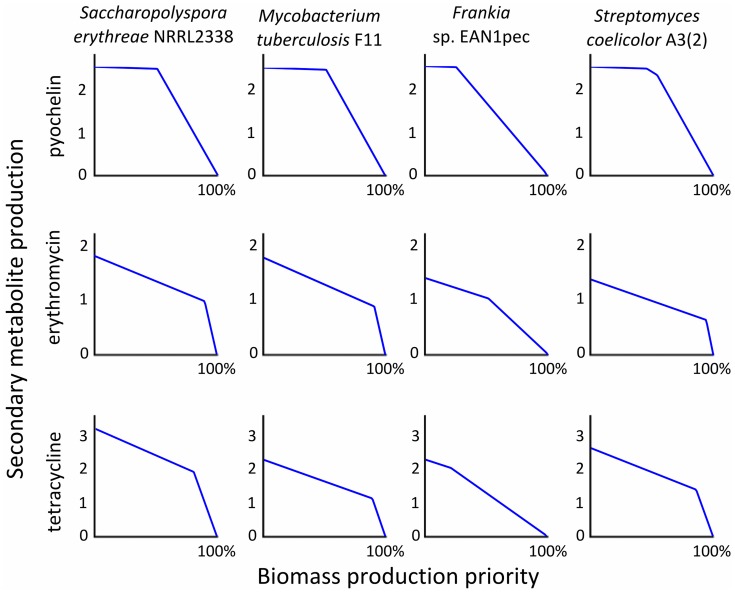
Pareto front calculation between biomass production and secondary metabolite biosynthesis. Pareto fronts are given for four species and three different natural products. To estimate secondary metabolite production, the flux rate through the final step in the biosynthetic pathway of the corresponding compound was used as a proxy.

In **Figure 4**, the y-axis on each plot represents the flux rate through the final biosynthesis reaction in the pathway for production of the given compound. The x-axis on each plot represents the percentage of maximal biomass production flux achieved. The region underneath the blue line represents the space of feasible solutions.

The predictions suggest that the overlap between network resources needed for biomass production and compound production differs notably between species, even without taking into account the organism-specific biomass compositions. It is likely that this has to do with the rate with which the network topology of a species enforces pathway competition between the two objectives, and to which extent alternative pathways are available for both processes.

The applied multi-objective analysis thus characterizes organism-specific relationships between biomass production and compound biosynthesis. Methods such as OptKnock [52] can subsequently enable metabolic engineers to reach a position close to the identified Pareto front, by determining how the compound production objective can be optimized given a certain biomass rate by for example stoichiometrically forcing the strain to synthesize a target compound as a by-product of growth.

In the simulations for pyochelin, biomass production at biomass maintenance levels (the almost horizontal plateau at the beginning of the curves) hardly competes with compound production. It appears that in this case the production of biomass components from the medium leaves several metabolic resources unused at the point where the first nutrient limitation from the medium prevents higher biomass production. We confirmed this by recalculating the trade-off under several different medium conditions. Indeed, we observed that pyochelin production and biomass production were constrained by different nutrient limitations: orthophosphate and NH_3_ were the limiting medium ingredients, respectively. When medium influx bounds of these compounds were increased by 100% each, the horizontal plateau disappeared.

In that sense, there is a “free lunch” for compound production as long as it is limited by a different nutrient than biomass production is. Remarkably, this suggests that production titers of industrial strains can sometimes be optimized without costs to the biomass maintenance.

In most plots, a single transition point is observed, at which the production titre starts to drop much more drastically when biomass production is increased. This might signify that the metabolic networks of these microbes have at least two distinguishable states in which a different nutrient is limiting for compound production given the fixed biomass production flux at that point. A “metabolic switch” seems to operate at this point, at which the regulation of metabolism probably needs to be drastically changed to maintain optimal levels of both biomass and compound production (i.e., to remain near the Pareto front). Of course, switch-like behaviour would be expected given that FBA is based on linear programming, and different linear constraints will be limiting at different points in the graph. Nonetheless, the fact that the switches corroborate observations from experimental microbiology, in which a carefully regulated switch has been observed at the onset of secondary metabolite biosynthesis [31], [53], suggests that cells may employ regulatory mechanisms to remain very close to such a theoretical polygon-shaped Pareto front [26].

### Conclusions

Comparative metabolic modelling is a new field, and as with any recent advance in biology, new software solutions are needed to achieve its full potential. With MultiMetEval, we provide an easy-to-use software framework to analyse large collections of metabolic models in parallel and to perform multi-objective analysis, coupled to the SurreyFBA framework. Although this is just a starting point for further software development, the tool already allowed us to study secondary metabolism in actinobacteria in novel ways. Most interestingly, comparative analysis of their genome-scale models predicts that the organisms whose genomes encode the largest numbers of biosynthetic gene clusters do not necessarily have the metabolic network topology most suited for industrial production of these compounds, suggesting an interesting line of enquiry for future experimental work. Additionally, results from multi-objective analysis suggest that bacterial metabolic switches are not just enforced by regulation, but are grounded in the very architecture of the metabolic system in which they occur. We expect that further experimental analysis will likely give exciting definitive insights into these phenomena.

## Supporting Information

Table S1 **Used methods for integration of KEGG pathways towards the biosynthesis of secondary metabolites.** To integrate the KEGG pathways for secondary metabolite biosynthesis in all 38 actinobacterial models, compound-specific Python scripts were written which used our in-house PyModelEditor to edit the models in such a way that they would allow simulation of compound biosynthesis. For the fifteen compounds chosen, different modifications had to be made to the models, as indicated in this table. The minimal medium used for flux balance analysis (FBA) consisted of H_2_O (influx upper bound 10000), O_2_ (10000), glucose (10), NH_3_ (10), PO_4_^3−^ (10), SO_4_^2−^ (10), CO_2_ (10), H^+^ (10), Cu^2+^ (10), Pb^2+^ (10), Zn^2+^ (10), Mn^4+^ (10), CrO_4_^2−^ (10), Mg^2+^ (10), K^+^ (10), Co^2+^ (10), Ca^2+^ (10), Fe^2+^ (10), Fe^3+^ (10), Cl^−^ (10), Ni^2+^ (10), Na^+^ (10), Cd^2+^ (10). Also, low influx of octadecanoic acid (0.001) was allowed, which was necessary to ‘start up’ some essential biosynthesis reactions.
(DOCX)

Table S2 **Correlation between reaction presence/absence and maximum fluxes.** For each compound, this table shows the squared correlation coefficient (r^2^) of the absence/presence of reactions in a metabolic model and the maximum flux obtained during flux balance analysis. The reaction name is the name as it is given in the SEED database, and the reaction formula corresponding to each reaction is given in the third column.
(XLSX)
