# Supplementary material for: MultiMetEval: Comparative and Multi-Objective Analysis of Genome-Scale Metabolic Models
Source: PLoS One. 2012 Dec 14;7(12):e51511. doi: 10.1371/journal.pone.0051511 (PMC3522732; doi:10.1371/journal.pone.0051511)
Supplement: Table S1 — Used methods for integration of KEGG pathways towards the biosynthesis of secondary metabolites. To integrate the KEGG pathways for secondary metabolite biosynthesis in all 38 actinobacterial models, compound-specific Python scripts were written which used our in-house PyModelEditor to edit the models in such a way that they would allow simulation of compound biosynthesis. For the fifteen compounds chosen, different modifications had to be made to the models, as indicated in this table. The minimal medium used for flux balance analysis (FBA) consisted of H2O (influx upper bound 10000), O2 (10000), glucose (10), NH3 (10), PO4 3− (10), SO4 2− (10), CO2 (10), H+ (10), Cu2+ (10), Pb2+ (10), Zn2+ (10), Mn4+ (10), CrO4 2− (10), Mg2+ (10), K+ (10), Co2+ (10), Ca2+ (10), Fe2+ (10), Fe3+ (10), Cl− (10), Ni2+ (10), Na+ (10), Cd2+ (10). Also, low influx of octadecanoic acid (0.001) was allowed, which was necessary to ‘start up’ some essential biosynthesis reactions. (DOCX) [file pone.0051511.s001.docx]

| **Compound** | **Modifications made** |
| --- | --- |
| All compounds | - Added glycolysis reactions (SEED IDs rxn05226, rxn00558, rxn00545, rxn00786) when missing, to allow glucose uptake and catabolism - Added methionine biosynthesis reactions when missing (SEED IDs rxn00952, rxn00953, rxn01300, rxn01301, rxn01302, rxn01303, rxn00693, rxn10113, rxn05256, rxn05902, rxn09240), based on Kovaleva & Gelfand, 2006. - Removed metabolite ‘Acyl_carrier_protein_c’ from the pathway, as a synthesis reaction for this metabolite is often missing. - Made KEGG pathway reactions irreversible to avoid flux block by looping |
| Aureomycin | - Added KEGG pathway rn00253 (R04060, R09191, R09197, R05456, R00742, R09198, R05459, R09194, R06641, R06642, R09187, R09188, R09195, R09189, R05463, R05462, R09192, R09196, R09199, R09200, R09193, R09190) - Made S-Adenosyl-L-homocysteine secretable, as its buildup would otherwise block fluxes |
| Butirosin | - Added necessary reactions from KEGG pathway rn00524 (R08911, R08617, R08890, R08892, R08891, R08894, R08895, R08896, R08897, R08898, R08899, R08905, R08907, R08906, R08908, R05705, R08909, R08910, R08893) - Added reaction for the conversion from 2-oxoglutaramate + H_2_O to 2-oxoglutarate + NH_3_ (KEGG R00269) - Made 5-oxoproline secretable, as its buildup would otherwise block fluxes |
| Cephalosporin | - Added KEGG pathway rn00311 (R07402, R03063, R07400, R02170, R06363, R03062, R05228, R03064, R05230, R05229, R05301, R06361, R04147, R04872, R04870, R05303, R07401, R05302) - Added alpha-aminoadipate biosynthesis reactions (SEED IDs rxn00310, rxn02916, rxn01578, rxn06127, rxn01664, rxn02226) when missing (based on Madduri et al., 1991). - Made AMP secretable, as its buildup would otherwise block fluxes |
| Clavulanic acid | - Added KEGG pathway rn00331 (R05465, R05467, R05471, R05469, R05468, R05357, R05466, R05470) - Removed orphan metabolite 5-oxoproline |
| Clorobiocin | - Added necessary reactions from KEGG pathway rn00401 (R06749, R06746, R06747, R06775, R06774, R06773, R06776, R06766, R06750, R06753, R06752, R06751, R06759, R06758, R01728, R00734, R06757, R06764, R06765, R06748) - Added sugar unit biosynthesis steps and essential dTTP/dTDP-converting reactions when missing (SEED IDs rxn00704, rxn01997, rxn02000, rxn02003, rxn01675, rxn01143) - Removed reaction rxn00527, which is a duplicate of a KEGG pathway rn00401 reaction - Made S-Adenosyl-L-homocysteine and dTDP secretable, as their buildup would otherwise block fluxes |
| Coumermycin | - Added necessary reactions from KEGG pathway rn00401 (R06768, R06767, R06778, R06777, R06749, R06746, R06747, R06750, R06755, R06754, R06753, R06752, R06751, R01728, R00734, R06756, R06748) - Added conversion reactions to form 3-Methylpyrrole-2,4-dicarboxylic acid, CO_2_ and orthophosphate from L-threonine and oxaloacetate (based on Siebenberg et al., 2011) - Added sugar unit biosynthesis steps and essential dTTP/dTDP-converting reactions when missing (SEED IDs rxn00704, rxn01997, rxn02000, rxn02003, rxn01675, rxn01143) - Made S-Adenosyl-L-homocysteine secretable, as its buildup would otherwise block fluxes |
| Enterobactin | - Added KEGG pathway rn01053 (R01505, R06602, R01717, R03037) - Added enterobactin biosynthesis reaction from 3.0 ATP, 3.0 L-serine and 3.0 2-3-dihydroxybenzoate to 3.0 ADP and 1.0 enterochelin/enterobactin (based on Reimman et al., 2001) - Added essential pyruvate-converting step (SEED ID rxn00499) - Made pyruvate secretable, as its buildup would otherwise block fluxes |
| Erythromycin | - Added KEGG pathway rn00522 (R06499, R06498, R06475, R06503, R06497, R06496, R06489, R06491, R06490, R06479, R06457, R06456, R06454, R06464, R06460, R05530, R02858, R02859, R06452, R06451, R06449, R06450, R06477, R06488, R06473, R06470, R06467, R05522, R06462, R06506, R05532, R06461, R06455, R05520, R05270, R00918, R06463, R05531, R05521) - Added polyketide sugar biosynthesis KEGG pathway rn0523 (R06513, R02328, R06428, R06424, R02773, R08583, R06427, R06426, R06443, R06436, R06429, R06435, R06433) - Added reactions for methylmalonyl-CoA biosynthesis, one from 1.0 succinyl-CoA to 1.0 methylmalonyl-CoA and one from 1.0 propanoyl-CoA and 1.0 CO_2_ to 1.0 methylmalonyl-CoA (KEGG R05373), based on Gross et al., 2006. - Added reaction for propanoyl-CoA biosynthesis where missing (SEED ID rxn04794) - Added reactions for D-glucose-1P biosynthesis where missing (SEED IDs rxn02302, rxn00704) - Made S-Adenosyl-L-homocysteine secretable, as its buildup would otherwise block fluxes |
| Neomycin | - Added necessary reactions from KEGG pathway rn00524 (R08617, R08890, R08892, R08891, R08894, R08895, R08896, R08897, R08898, R08899, R08900, R08901, R08903, R08893, R08902, R08904) - Added reaction for the conversion from 2-oxoglutaramate + H_2_O to 2-oxoglutarate + NH_3_ (KEGG R00269) - Made 5-oxoproline secretable, as its buildup would otherwise block fluxes |
| Novobiocin | - Added necessary reactions from KEGG pathway rn00401 (R06750, R06772, R06771, R06770, R06769, R06755, R06754, R06753, R06752, R06751, R06759, R06758, R01728, R00734, R06757, R06756) - Added conversion reactions to form 3-Methylpyrrole-2,4-dicarboxylic acid, CO_2_ and orthophosphate from L-threonine and oxaloacetate (based on Siebenberg et al., 2011) - Added sugar unit biosynthesis steps and essential dTTP/dTDP-converting reactions when missing (SEED IDs rxn00704, rxn01997, rxn02000, rxn02003, rxn01675, rxn01143) - Made S-Adenosyl-L-homocysteine secretable, as its buildup would otherwise block fluxes |
| Penicillin | - Added KEGG pathway rn00311 (R07402, R03063, R07400, R02170, R06363, R03062, R05228, R03064, R05230, R05229, R05301, R06361, R04147, R04872, R04870, R05303, R07401, R05302) - Added alpha-aminoadipate biosynthesis reactions (SEED IDs rxn00310, rxn02916, rxn01578, rxn06127, rxn01664, rxn02226) when missing (based on Madduri et al., 1991). - Made AMP secretable, as its buildup would otherwise block fluxes |
| Pyochelin | - Added KEGG pathway rn01053 (R01505, R06602, R01717, R03037) - Added pyochelin biosynthesis reaction from 2.0 L-cysteine + 1.0 salicylate + 1.0 S-adenosyl-L-methionine + 3.0 ATP + 1.0 NADPH + 1.0 H_2_O to 1.0 pyochelin + 1.0 S-adenosyl-L-homocysteine + 3.0 ADP + 1.0 NAD (based on Reimman et al., 2001) - Made S-Adenosyl-L-homocysteine secretable, as its buildup would otherwise block fluxes |
| Streptomycin | - Added necessary reactions from KEGG pathway rn00521 (R03384, R01187, R07324, R02777, R02225, R02228, R04222, R06514, R06513, R02328, R08639, R03477, R05516, R05515, R05512, R06365, R01183, R02781, R08843, R08844, R05547, R03496, R03502) - Added reaction for the conversion from 2-oxoglutaramate + H_2_O to 2-oxoglutarate + NH_3_ - Removed orphan metabolites ‘Amino_group_donor_c’ and ‘Activated_methyl_group_c’ - Added sugar unit biosynthesis steps and essential dTTP/dTDP-converting reactions when missing (SEED IDs rxn00704, rxn01997, rxn02000, rxn02003, rxn01675, rxn01143) - Made dTDP secretable, as its buildup would otherwise block fluxes |
| Tetracycline | - Added KEGG pathway rn00253 (R04060, R09191, R09197, R05456, R00742, R09198, R05459, R09194, R06641, R06641, R06641, R06642, R09187, R09188, R09195, R09189, R05463, R05462, R09192, R09196, R09199, R09200, R09193, R09190) - Made S-Adenosyl-L-homocysteine & NAD+ secretable, as their buildup would otherwise block fluxes |
| Tylosin | - Added KEGG pathway rn00522 (R06499, R06498, R06475, R06503, R06497, R06496, R06489, R06491, R06490, R06479, R06457, R06456, R06454, R06464, R06460, R05530, R02858, R02859, R06452, R06451, R06449, R06450, R06477, R06488, R06473, R06470, R06467, R05522, R06462, R06506, R05532, R06461, R06455, R05520, R05270, R00918, R06448, R06463, R05531, R05521) - Added polyketide sugar biosynthesis KEGG pathway rn0523 (R06439, R06513, R02328, R06428, R06424, R06423, R02773, R08583, R06427, R06426, R06443, R06438, R06437, R06436, R06429, R06435, R06433) - Added reactions for methylmalonyl-CoA biosynthesis, one from 1.0 succinyl-CoA to 1.0 methylmalonyl-CoA and one from 1.0 propanoyl-CoA and 1.0 CO_2_ to 1.0 methylmalonyl-CoA (KEGG R05373), based on Gross et al., 2006. - Added reactions for ethylmalonyl-CoA biosynthesis (based on KEGG R09291 and R03027) - Added reaction for propanoyl-CoA biosynthesis where missing (SEED ID rxn04794) - Added reactions for D-glucose-1P biosynthesis where missing (SEED IDs rxn02302, rxn00704) - Made S-Adenosyl-L-homocysteine secretable, as its buildup would otherwise block fluxes |
